# Assessment of the Etiologies and Outcomes of Antenatal Hydronephrosis in Patients at King Abdulaziz University Hospital

**DOI:** 10.7759/cureus.7615

**Published:** 2020-04-10

**Authors:** Osama Safdar, Mohammed A Safhi, Omar Saggaf, Hassan R Algethami, Mohammed Alhalabi, Eyad M Alnajrani, Ahmed Baeshen, Marwan Filemban

**Affiliations:** 1 Pediatrics, King Abdulaziz University, Jeddah, SAU; 2 Surgery, Faculty of Medicine, King Abdulaziz University, Jeddah, SAU; 3 Internal Medicine, King Abdulaziz University, Jeddah, SAU; 4 Medicine, King Abdulaziz University, Jeddah, SAU; 5 Emergency Medicine, Faculty of Medicine, King Abdulaziz University, Jeddah, SAU; 6 Pediatrics, Faculty of Medicine, King Abdulaziz University, Jeddah, SAU

**Keywords:** antenatal hydronephrosis, outcome, congenital anomalies, vesicoureteral reflux, urinary tract infection, prognosis

## Abstract

Background

Antenatal hydronephrosis (ANH) is the most common congenital abnormality. It is often detected during pregnancy through an antenatal ultrasound (US) examination. This condition is defined as the dilatation of the renal pelvis and/or calyces in neonates. Little is known about ANH and its associated etiology and outcomes, especially in the Middle East. This study aims to determine the outcome of patients diagnosed with ANH between 2011 and 2017.

Methods

The current study was a retrospective analysis of data collected from the medical records of 64 ANH patients (45 male, 19 female). We collected data regarding the demographic characteristics, fetal parameters, laboratory and radiological parameters, and medical and surgical interventions. Moreover, based on radiological reports patients were classified into one of the following two groups: good prognosis, including patients with improved or resolved ANH and poor prognosis, including patients with progressing ANH and associated complications such as parenchymal scarring, chronic kidney disease complicated by secondary hypertension.

Results

Overall, 48.4% of patients exhibited good prognosis, whereas 51.6% exhibited poor prognosis. Among the 64 patients, 53.1% of patients exhibited unilateral ANH, and 46.9% exhibited bilateral ANH. Also, unilateral ANH and bilateral ANH had good prognoses in 41.2% and 56.7%, respectively, while unilateral and bilateral ANH had poor prognosis in 58.8% and 43.3%, respectively.

Conclusions

Additional prophylactic measures should be taken to avoid complications, such as urinary tract infection (UTI), as our study found that patients who have ANH are more prone to develop UTI. Patients with several ANH comorbidities are associated with poor prognosis.

## Introduction

Congenital anomalies in the kidney and urinary tract (CAKUT) are a severe set of disorders that are related to a number of factors, including incomplete nephron development, malformation of renal parenchyma, improper embryonic migration of the kidneys and urinary tract, and abnormal development of the collecting system [1]. Amongst all fetal congenital anomalies, CAKUT makes up roughly 30%, with the most common conditions being antenatal hydronephrosis (ANH), polycystic kidney disease, multi-cystic dysplastic kidney, and renal agenesis [1-2].

ANH is one of the most common abnormalities during pregnancy; it is commonly detected during antenatal ultrasound examinations [3]. This condition is defined as a dilatation of the renal pelvis and/or calyces in neonates and is noted in approximately 0.5%-1% of pregnancies [3]. Several systems have been developed for the diagnosis and grading of ANH severity [4-5]. For example, the Society of Fetal Urology (SFU) criteria depend on the renal pelvic diameter (RPD), renal calyceal involvement, and renal parenchymal atrophy [6-7]. Another grading system included the RPD, which is a measure of the widening and/or dilation of the renal pelvis and is classified as mild (5-9 mm), moderate (10-15 mm), or severe (> 15 mm) [6,8-11]

Recent studies have reported that ANH is the most common antenatal detected urinary tract abnormality and that in most cases, ANH is resolved within two months after birth [12]. However, this finding was challenged by other research suggesting that there is no consensus on the classification and management of ANH to date [8,13]. One of the current controversies stems from the fact that it is difficult to determine whether this condition is pathological or transient on US imaging [10]. While most cases of ANH resolve spontaneously, the inability to predict the outcome can cause caregivers extreme concern, which could be easily avoidable by early testing. The early detection of persistent ANH may be essential to prevent the progression of renal damage [10,14]. The effects of untreated ANH are manifested as an initial increase in intratubular pressure, progressing to the compression of renal blood vessels, and culminating in decreased renal perfusion, ischemic tubular atrophy, and thinning of renal cortex and medulla; all these factors contribute to irreversible loss of renal function [4].

Unfortunately, little is currently known about ANH and its associated etiology and outcomes. Due to the distress associated with this disorder, it is imperative to predict the outcomes and to detect the condition during the antenatal stage to determine prognosis as well as the best course of treatment. This study aims to determine the outcome of patients diagnosed with ANH between 2011 and 2017.

## Materials and methods

The current retrospective study included 83 patients, 19 of whom were excluded due to loss of follow-up. The remaining 64 patients (45 males, 19 females) were treated for ANH at King Abdul-Aziz University Hospital between 2011 and 2017. Data were collected from the hospital records. We recorded demographic data, fetal parameters, treatment data, laboratory and radiology reports, and patient outcomes. According to the radiological report on the follow-up, the patients were categorized into one of the following two groups: good prognosis, including patients whose ANH improved or resolved, and poor prognosis, including patients whose ANH progressed and resulted in associated complications, such as parenchymal scarring and chronic kidney disease (CKD) with hypertension (HTN).

Statistics

Statistical data were analyzed using IBM SPSS software (Version 23 for Windows; IBM, Armonk, NY). The chi-square and Fisher's exact tests were applied, when appropriate, for comparisons between groups to determine significance. All p-values of <0.05 were considered statistically significant.

## Results

Gender and ANH presentation

Of the 64 patients with ANH finally included in the current study, 70.3% were male and 29.7% were female. In terms of ANH prognosis, 31 patients (48.4%) had a good prognosis and 33 patients (51.6%) had a poor prognosis. Despite the unequal gender ratio, gender was not shown to be a significant factor between good and poor ANH classifications (chi-square test value = 0.148, p = 0.700). There was no significant difference between the unilateral and bilateral ANH presentations; 53.1% of patients exhibited unilateral ANH and 46.9% exhibited bilateral ANH. Among patients with unilateral ANH, 41.2% had a good prognosis and the remaining 58.8% had a poor prognosis. Among patients with bilateral ANH, 56.7% had a good prognosis and the remaining 43.3% had a poor prognosis. Testing revealed that there was no significant difference in ANH-associated prognoses between unilateral and bilateral ANH (chi-square test value = 0.974, p = 0.324). Among patients with unilateral ANH, the current data indicate a possible trend of the left kidney (73.5%) being more impacted than the right kidney (26.5%; chi-square test value = 0.907, p = 0.341), although the data are not statistically significant.

ANH comorbidities and prognosis

Analysis of the associated conditions amongst the good and poor prognosis groups revealed that among the four patients (6.3%) with both ANH and polycystic kidney disease, two (50.0 %) were classified in the good prognosis group and the other two (50.0 %) were classified in the poor prognosis group. Among the three (4.7%) with both ANH and a neurogenic bladder, all were classified in the poor prognosis group. Among the patients without a neurogenic bladder, 50.8% were classified in the good prognosis group.

With respect to patients with several ANH comorbidities, the prognosis was shown to be especially affected (Tables 1-2). For example, among the five patients who experienced urinary tract infections (UTIs), four had a good prognosis and only one had a bad prognosis. Amongst the six patients who exhibited both a UTI and vesicoureteral reflux (VUR), five had a poor prognosis and only one had a good prognosis. Among the four patients with UTI, VUR, and ureteropelvic junction obstruction (UPJO), all four had a poor prognosis. Furthermore, when only considering the 20 patients who had a UTI alone or a UTI with one or several complications, including VUR and UPJO, those exhibiting a UTI with one or more complication had significantly poorer prognoses than did those who had UTI alone (chi-square test value = 10.168, p = 0.017; Tables 1-2). Taken together, these data suggest a relationship between ANH with UTI, VUR, and UPJO comorbidities and the resulting prognosis.

**Table 1:** ANH-associated comorbidities and the impact upon patient prognosis The first column depicts the specific ANH-associated comorbidity. The second column indicates whether or not the comorbidity affected the participants. The third and fourth columns depict the percentage of participants classified with either good or poor prognosis. Data are shown as the number of patients and the percentage of the total participant group. The fifth column depicts the corresponding p-value measuring comorbidity affected and prognosis type. p-values <0.05 were considered statistically significant. a: Fisher's exact test; b: Pearson chi-square test; ANH: antenatal hydronephrosis

ANH-associated comorbidity		Good prognosis	Poor prognosis	p-value
Polycystic kidney	Yes	2 (6.5%)	2 (6.1%)	1^a^
	No	29 (93.5%)	31 (93.9%)	
Neurogenic bladder	Yes	0 (0%)	3 (9.1%)	0.239^a^
	No	31 (100%)	30 (90.9%)	
Down syndrome	Yes	1 (3.2%)	1 (3%)	1^a^
	No	30 (96.8%)	32 (97%)	
Urinary tract infection	Yes	9 (29%)	11 (33.3%)	0.711^b^
	No	22 (71%)	22 (66.7%)	
Ureteropelvic junction obstruction	Yes	16 (51.6%)	14 (42.4%)	0.462^b^
	No	15 (48.4%)	19 (57.6%)	
Vesicoureteral reflux	Yes	5 (16.1%)	16 (48.5%)	0.006^b^
	No	26 (83.9%)	17 (51.5%)	

**Table 2:** UTI-associated ANH comorbidities The first column depicts UTI comorbidities. The second and third columns depict the number of participants classified in the good or bad prognosis groups. The fourth column depicts the number of total participants for each UTI associated comorbidity including both prognosis groups. UTI: urinary tract infection; UPJO: ureteropelvic junction obstruction; VUR: vesicoureteral reflux

UTI and associated conditions	Good prognosis	Bad prognosis	Total
UTI Only	4	1	5
UTI with VUR Only	1	5	6
UTI with UPJO Only	4	1	5
UTI with UPJO and VUR	0	4	4
Total	9	11	20

ANH treatment and prognosis

Of patients who did not undergo any surgical intervention, 13 (52%) were shown to have a better prognosis while 12 (48%) had a worse prognosis. In contrast, of the patients who underwent one or more surgeries, 21 (53.8%) had worse prognosis and 18 (46.2%) had a better prognosis (non-significant; chi-square test value = 0.040, p = 0.841). No significant effect on prognosis was found with respect to the administration of antihypertensive drugs, vitamin D, iron, sodium bicarbonate, calcium, oxybutynin, or antibiotic prophylaxis (Table 3).

**Table 3:** Medication usage among the included study patients The medication type is listed on the left-hand column. The second column indicates whether or not a particular medication type was administered. The third and fourth columns depict the percentage of participants classified with either a good or a poor prognosis. The fifth column depicts the corresponding p-value measuring medication usage and prognosis type. p-values <0.05 were considered statistically significant. a: Fisher's exact test; b: Pearson chi-square test

Type of medications		Good prognosis	Poor prognosis	p-value
Antihypertensive drugs	Yes	9.7%	21.2%	0.305^a^
	No	90.3%	78.8%	
Vitamin D	Yes	22.6%	33.3%	0.498^b^
	No	77.4%	66.7%	
Iron	Yes	22.6%	42.4%	0.114^a^
	No	77.4%	57.6%	
Sodium bicarbonate	Yes	12.9%	18.2%	0.734^a^
	No	87.1%	81.8%	
Oxybutynin	Yes	29%	30.3%	1.0^b^
	No	71%	69.7%	
Calcium	Yes	3.2%	15.2%	0.198^a^
	No	96.8%	84.8%	
Antibiotics prophylaxis	Yes	87.1%	97%	0.190^a^
	No	12.9%	3%	

## Discussion

We reviewed patient medical records to determine ANH outcomes when various complications are present. Previous work has indicated similar rates of antenatally detected renal abnormalities. For example, a recent study reported a rate of 1.7%, while earlier studies reported rates of 0.5%-1% [5,7,12,15-17]. The slight variation in the reported occurrence rates might indicate the potential influence of outside factors. For example, the occurrence rate can vary between 0.6% and 5.4% when different geographic regions are studied [17-20].

A variety of associated ANH outcomes have been reported in previous works, ranging from poor prognosis to spontaneous and complete recovery [21-24]. This range has been indicated to relate to ANH severity; previous works have demonstrated that between 78% and 96% of mild ANH cases result in spontaneous recovery [5,8,12,16,25]. The current study findings are consistent with that of a previously published study showing that ANH resolved in 41.2% of patients and reached a good prognosis within two months with a value of 48.4% of participants exhibited good prognosis [12]. Altogether, our study and the studies by Kari et al. and Chaudhary and Shah suggest that the outcome is possibly affected by the severity of ANH [12,26].

The current study found that 41.2% of the unilateral and 56.7% of the bilateral ANH patients had a good prognosis, while 58.8% of the unilateral and 43.3% of the bilateral ANH patients had a poor prognosis. Although this could be potentially connected to VUR, 12 (35.3%) had unilateral VUR, and only nine (30%) patients had bilateral VUR. However, there was no statistically significant difference.

The finding that there was a relationship between patients who exhibited a UTI, with one or more associated conditions, such as VUR, and UPJO, and ANH prognosis could indicate that the presence of multiple ANH-associated comorbidities may increase the burden on health and thereby decrease overall ANH outcomes. Nevertheless, it is important to note that a previous study reported a poor correlation between VUR grade and ANH severity, suggesting that there might be nuances to this connection, which have yet to be discovered [26]. It is possible that including VUR grading into the current study would have yielded more definitive results and addressed this potential discrepancy. Future studies on ANH should include this analysis to improve the efficacy of experimental results.

Concerning gender characteristics among the current study participants, the male-to-female ratio of 2.4:1 was expected. Also, previous research showed that ANH is more common in males [8,12,14,25-26].

With respect to study limitations, a major shortcoming of the current study was the fact that there was data missing from the patient files. For example, information such as ‎height, glomerular filtration rate (GFR), pelvic diameter, and perinatal history was not reported for the majority of study participants, limiting the scope of the current findings. Moreover, the small sample size also limited the current study results.

Future research would benefit from increasing the sample size and including patients from several hospitals. Other important factors to consider include assessing the potential impact of factors and diseases that could affect the mother during pregnancy. For example, it would be interesting to investigate amniotic fluid and the chances of an obstruction such as oligohydramnios to occur during pregnancy. Furthermore, an exploration of how gender and ethnicity might impact ANH could provide compelling results. Finally, an investigation into various medications and their effectiveness in supporting patient health and associated ANH outcomes would also be an interesting avenue for future ANH-associated research.

## Conclusions

We found that amongst the included participants, 48.4% had a good prognosis and 51.6% had a poor prognosis. Our data also suggest that the ANH prognosis is related to both the degree and etiology of ANH. Participants with a lower grade of ANH were less likely to develop a UTI or undergo surgical intervention, whereas infants with a higher ANH grade were more likely to develop a UTI and undergo surgical interventions associated with poor prognosis.

## Appendices

**Table 4:** Patient outcomes depict unilateral and bilateral ANH The first column depicts patient outcomes. The second column depicts the number of participants in each outcome. The third column illustrates the number of participants in bilateral ANH. The fourth column describes the number of participants in unilateral ANH. ANH: antenatal hydronephrosis; CKD: chronic kidney disease, HTN: hypertension

Patient outcomes	Affected patients	Bilateral	Unilateral
Improved	28 (43.8%)	15(50%)	13(38.2%)
Resolved	3 (4.7%)	2(6.7%)	1(2.9%)
Progressed or persistent	23 (35.9%)	5(16.7%)	18(52.9%)
CKD with HTN	4 (6.3%)	3(10%)	1(2.9%)
Death	4 (6.3%)	4(13.3%)	0
parenchymal scarring	2 (3.1%)	1(3.3%)	1(2.9%)
Total	64	30	34

**Table 5:** RPD classification among the included study patients RPD classification is listed in the left-hand column. The second and third columns depict the number and percentage of participants classified among a right or a left kidney. RPD: renal pelvic diameter

Grade	Right Kidney	Left Kidney
Mild (5-9 mm)	14 (35.9%)	19 (34.5%)
Moderate (10-15 mm)	7 (17.9%)	13 23.6%)
Severe (> 15 mm)	18 (46.2%)	23 (41.8%)
Total	39	55
